# The effects of Botulinum toxin on the detection of gradual changes in facial emotion

**DOI:** 10.1038/s41598-019-48275-1

**Published:** 2019-08-13

**Authors:** L. C. Bulnes, P. Mariën, M. Vandekerckhove, A. Cleeremans

**Affiliations:** 1 Université libre de Bruxelles (ULB), Consciousness, Cognition and Computation Group (CO3), Center for Research in Cognition & Neurosciences, ULB Neuroscience Institute, Brussels, Belgium; 2 Vrije Universiteit Brussel (VUB), Biological Psychology Research Unit (BIPS), Brussels, Belgium; 3 Vrije Universiteit Brussel (VUB), Clinical and Experimental Neurolinguistics, Brussels, Belgium; 4 ZNA Middelheim Hospital, Department of Neurology and Memory Clinic, Antwerp, Belgium

**Keywords:** Perception, Neurophysiology, Human behaviour

## Abstract

When we feel sad or depressed, our face invariably “drops”. Conversely, when we try to cheer someone up, we might tell them “keep your smile up”, so presupposing that modifying the configuration of their facial muscles will enhance their mood. A crucial assumption that underpins this hypothesis is that mental states are shaped by information originating from the peripheral neuromotor system — a view operationalised as the Facial Feedback Hypothesis. We used botulinum toxin (BoNT-A) injected over the frown area to temporarily paralyse muscles necessary to express anger. Using a pre-post treatment design, we presented participants with gradually changing videos of a face morphing from neutral to full-blown expressions of either anger or happiness and asked them to press a button as soon as they had detected any change in the display. Results indicate that while all participants (control and BoNT-A) improved their reaction times from pre-test to post-test, the BoNT-A group did not when detecting anger in the post-test. We surmise that frown paralysis disadvantaged participants in their ability to improve the detection of anger. Our finding suggests that facial feedback causally affects perceptual awareness of changes in emotion, as well as people’s ability to use perceptual information to learn.

## Introduction

A key hypothesis about the mind-body relationship is that information coming from the periphery and the neuromotor system influences both behaviour and subjective experience^1^. Intuitively, when humans feel down, they do not show the posture or the face of a winning warrior. Rather, their face inevitably “drops”. Conversely, when we try to cheer someone up, we might tell them to keep smiling, implying that modifying the configuration of the facial muscles will enhance their mood. These observations suggest a bidirectional relationship between emotional states and facial expressions where the body plays a central role in shaping subjective experience. From this perspective, bodily information, in the form of reafferent signals, is thus crucial to achieve a stable and correct perception of the environment^2^. Von Helmholtz, in the 19^th^ century, already proposed the idea that whenever a motor command is issued, a parallel feedforward predicted sensory signal is also triggered (the efference copy). This predicted signal is then compared to the actual afferent information that resulted from the generated action. The *feedback* generated by proprioception coming from muscles, joints, and skin, also called “reafference”, is compared to an internal, expected signal, and constitutes an inferential process that guides awareness and subjective experience. In keeping with this neurophysiological account of the perception-action relationship, the production of an emotional facial expression feeds back to the central nervous system (CNS), so contributing to modulate or initiate the subjective experience of emotion (for a review see ref.^1^).

This interactive view was also articulated by Darwin, who posited that “the outward signs of an emotion intensifies it”. The core assumption of Darwin’s proposal is that facial feedback information plays a causal role in the subjective experience of emotion^1,3^. Facial mimicry, in particular, is assumed to mediate this process^4,5^ (but see ref.^6^ for contrasting results) and to subserve empathy and emotion understanding^7^.

This idea that facial mimicry is central to the perception of emotion was echoed by Fritz Strack through his work on the “facial feedback hypothesis”. In a landmark article (>1000 citations), Strack *et al*.^8^ showed that people asked to judge how funny cartoons are, gave higher ratings when holding a pen between their teeth vs. between their lips. Strack *et al*.^8^ interpreted this striking result by assuming that people’s subjective experience of the cartoons is affected by the configuration of their facial musculature, induced by the manner in which the pen is held: When holding a pen between the front teeth, the facial muscles are put in the configuration they have when one smiles; whereas holding a pen between the lips activates facial muscles (e.g., the orbicularis oris) whose activity is incompatible with smiling. Just as perception influences action, there is a loop that extends back from action to perception. Similarly, Larsen, *et al*.^9^ showed that when participants were asked to hold two golf tees in the frown area and asked to either touch the tips of the golf tees together or to hold them apart, they rated negative pictures as more unpleasant in the “touch them together” compared to the “hold them apart” condition, giving further support to the facial feedback hypothesis.

However, as sound as it may appear, the idea that our emotional states are modulated by the state of our peripheral musculature has also been challenged. For instance, a recent Multilab (17 labs) OSF replication in which we took part^10^ failed to confirm the core finding of the famous Strack *et al*. study^8^. Yet, this failure –or the presence or absence of the effects- seemed attributable to a single variation in the design, as an even more recent study reported results supporting both, the famous Strack et al. study as well as the OSF replication report^11^. The crucial element being the presence or absence of a recording camera during the experiment. This leaves open many questions. Specifically, do changes in our facial expression of emotion genuinely influence our processing of emotions? If so, how does feedback from the face influences the perception and the experience of emotion?

Many studies in the electromyography literature have shown that mere exposure to facial expressions elicits fast (<500 ms from stimulus onset) activation of specific and congruent facial musculature^12,13^. Thus, zygomaticus activity is found when processing positive emotional faces, while the corrugator supercilii is activated when processing negative emotional faces (for a review see ref.^14^). This mimicry effect is so compelling that it does not even require the conscious perception of the stimulus, because it has also been demonstrated when static facial stimuli are presented at near-threshold level^15,16^. The effect further persists in cortical blindness (due to loss of primary visual cortex), in a condition known as blindsight —the striking ability of participants to discriminate at above chance-level stimuli they cannot consciously see^17^. The question, however, still remains that if facial feedback is crucial for emotional processing, what happens when one is not able to produce a given facial expression?

### Disruption of facial processing

Goldman and Sripada^18^ suggest that in virtue of a failure in mimicry, during a disruption of facial expression, the internal “simulation” of the emotion in question does not succeed, resulting in impaired emotion recognition. Congruently, recent findings show that the selective introduction of muscular noise in the zygomaticus muscle (ie. asking participants to bite a pen with their teeth and lips) produces a stronger N400 component during the processing of happiness, thus suggesting substantial semantic load during the processing of stimuli that required feedback from the lower face^19^. Furthermore, virtual lesion studies with Transcranial Magnetic Stimulation (TMS) have shown that neural disruption over the face right-somatosensory cortex and over the right occipital face area (rOFA) both selectively impair participants’ accuracy in a face expression discrimination task (e.g.^20^). In the same vein, TMS over somatosensory and motor areas corresponding to the face area seem to have clear disruptive effects in the processing of fear^21^, the mimicry of smiles in female participants^22^, and the judgment of the authenticity of smiles^23^.

These findings suggest that neural noise induced by TMS impairs the neural simulation of the somatovisceral and motor responses associated with the facial-emotional expressions and has direct consequences on spontaneous muscular responses to faces. Nevertheless, data from lesion and neuromuscular pathology studies have not always been consistent with this assumption. For example, the study of locked-in syndrome, which is characterised by a muscular facial paralysis of voluntary movement (pyramidal tract) due to a localised lesion in the pons, shows that patients with this condition are particularly impaired in facial emotion recognition as well as in expression intensity rating, but not when the task requires identification of natural emotional scenes^24^. In line with these results, patients presenting progressive supranuclear palsy (PSP), a parkinsonian syndrome that affects the ability to accurately display facial expressions, are impaired in the recognition of facial expression of emotions but do not display difficulties recognizing faces^25^. These impairments, however, are not observed when lesions occur early in life. For instance, studies about the Möbius syndrome, a very rare pathology characterised by the congenital malformation of cranial nerves 6 (facial motor of orbital area) and 7 (facial nerve, sensory & motor: main nerve for facial expressions), have shown that adults with this condition do not present facial expression recognition deficits, and concluded that facial feedback is not necessary for emotional expression processing^26,27^. These inconsistencies in the lesion literature have prompted different experimental studies that leveraged the use of botullinum toxin (BoNT-A) to enable the exploration of experimentally induced selective facial paralysis.

The BoNT-A toxin is a very powerful enzyme that causes flaccid muscular paralysis. It acts by cleaving the proteins responsible for liberating acetylcholine at the pre-synaptic space of the neuromuscular junction^28^. It weakens both voluntary (pyramidal) as well as involuntary (extrapyramidal) muscular processes (for a review see ref.^29^). Some studies using BoNT-A have reported that facial feedback appears necessary to process emotional stimuli. For instance, when the toxin is applied in the frown area, it has been shown that subjects undergo a change in how they perceive their emotional experience^30^. The putative mechanism involves a loop between action and perception, mediated by other people: Having a face that expresses fewer wrinkles in the frown area results in a less angry-looking face, which in turn makes other people perceive the face less negatively and hence respond more favourably to it. This, in turn, reinforces our own positive emotions and dimishes one’s likelihood to exhibit a wrinkled, sad, angry, or negative face. Beyond the effects of the cosmetic procedure on wellbeing^31^, recent findings show how BoNT-A injections on the glabella lines region (frown area) specifically affect the reading times of sentences with negative emotional valence compared to positive sentences^32^. Further, these effects on reading negative material seem to be more pronounced when the sentences are only mildly emotional and therefore need extra cognitive processing^33^. Conversely, two recent studies, one using the “Reading the Mind in the Eyes” paradigm^34^ and the other assessing perceived emotional experience after watching videos with emotional content^35^ both demonstrated a rather general effect of BoNT-A paralysis, suggesting a moderate influence of facial feedback in emotional processing. For instance, in the study of Davis *et al*.^35^, which involved a pre-post test design, participants who received the toxin rated positive videos as less intense, in contrast to a group that received filler gels applied to the nasolabial folds. However, in this study, BoNT-A was not only applied over the frown area, but also over the orbicularis oculi area, which is a key muscle to the successful expression of a Duchene smile. Furthermore, the results showed an overall decrease of the magnitude of emotional experience, which was specific to mildly positive clips (the study’s neutral condition). The Facial feedback hypothesis states that changes in facial expression induced through facial muscle changes (irrespective of what caused the change) may lead to changes in emotional states. However, this was not the case here, as most musculature necessary to produce a negative expression was altered, and musculature necessary to produce a genuine smile was only partially affected.

Likewise, in the Neal and Chartrand^34^ study, participants showed impairment in the ‘Reading the Mind in the Eyes’ task and were slower when compared to controls who had received dermal fillers. However, this pattern was reversed when a gel facemask was applied in the area corresponding to the RMET stimuli (eg. lower forehead, brow, and eye contour), allegedly via a mechanism of « proprioceptive feedback »^36^ that amplifies the afferent signal because of the local pressure exerted by the gel. However, it should be noted that the toxin was also applied beyond the frown area in this study, a limitation that was also present in the Baumeister et al.study^33^ and the Davis *et al*.^35^ study. This included the forehead and the crow’s feet area. Moreover, the control condition (dermal fillers), similar to the hardening gel, also creates dermal pressure. Therefore, it is difficult to assess the contributions of a possible enhancement (as with the hardening gel) insofar as a comparison with the Botox group is concerned. Notably, they did not perform a pre-post measure and the results did not show any procedure × valence interactions, perhaps due to the fact that several muscles had been compromised. The authors concluded that facial feedback merely plays a « moderator » role because information conveyed from the face is differentially processed depending on whether the signal is amplified of disrupted.

Interestingly, using functional neuroimaging with magnetic resonance (fMRI), Hennenlotter *et al*.^37^ showed that the deafferentation of frown muscles with BoNT-A selectively attenuates the intensity of contraction of the corrugator muscles, and underactivates amygdalar response when participants are overtly asked to imitate expressions of anger and sadness^37^. Thus the disruption created by deafferentation seems to directly modulate brain structures that help maintain and update the adaptive flow of affective experiences^38^. This result could explain why the flaccid paralysis of the frown area attenuates the positive symptoms of depression^39,40^ and migraine^41,42^, by dampening the negative experience related to depression and pain sensation. The finding that the capacity to produce a given facial expression (e.g. anger) is functionally related to key brain emotional centres (e.g. Amygdala) has been likewise observed when participants are passively viewing pictures of anger^43^. In the study by Kim *et al*.^43^ in a pre-post-return to baseline (A-B-A) experiment, the toxin was applied only in the frown area of each participant. The task consisted of deciding by a button press about the valence (i.e. positive or negative) of flashed static pictures of faces (17 ms, 50 ms, and 1000 ms). Notably, the results failed to show any behavioral effects, as assessed by the lack of difference on reaction times for both emotions across time. They demonstrated, however, that the amygdala was more active during the exposure to angry faces as compared to happy faces before deafferentation and once the effects of the toxin had subsided. Crucially, the study failed to show, strictu-sensu, that the deafferentation had any specific effects either in emotional experience or in awareness of facial expression. To our knowledge, the study of Havas *et al*.^32^ is the only study specifically targeting the frown area and demonstrating a detrimental effect of face deafferentation in negative emotional processing. However, their study investigated sentence reading times rather than face perception.

In sum, while previous literature has successfully shown a relationship between face expressive behavior and overall emotional processing, the specificity of the association between facial muscle activity and emotional experience or awareness remains inconclusive.

### The present study

Taken together, the findings obtained with BoNT-A mostly concern the effects of somatosensory disruption on the conceptual processing of facial expressions. Most tasks leveraged in previous studies using the toxin required explicit mentalizing. Indeed, the “Reading the Mind in the Eyes” task, tasks that require reports of emotional experience or judgments of emotional intensity, tasks that involve sentence comprehension, introspective reports, or even face categorisation all require deliberate and explicit processing of a target’s emotion, or intentions. Therefore, a great deal of conceptual processing was elicited. However, the literature about change detection paradigms demonstrates that the detection of changes in visual displays depends more on short-term memory representations than on long-term semantic knowledge^44–46^. As such it is likely that most previous literature exclusively probed an “as-if” account of somatosensory simulation rather than a read-out process^18^. In this respect, in a recent study by Wood *et al*.^47^, participants underwent the placement of a cosmetic peel-off mask over the entire face. In this procedure, the applied gel dries in minutes and becomes plastic, creating stiffness in the face area. Two tasks were administered while participants were being treated. First, participants carried out a perceptual discrimination task in which a target image was presented (700 ms), and presented again together with a distractor (e.g., two versions of the same target) after a short mask (300 ms). Participants were required to discriminate the correct target, which consisted of static pictures of morphed combinations of sadness and anger. Subsequently, subjects performed an identification task where, after a target was presented (750 ms), a forced choice was required by selecting one of two labels that best described the category of the target (e.g., sad - angry). Crucially, they reported that participants with the gel-mask performed worse than the controls on the visual-matching task for targets that involved facial expressions, but not in the identification task. These results demonstrate how a sensorimotor manipulation of the face causally affected perceptual processing of facial expressions. However, given that the mask was applied to the entire face area, it remains difficult to assess specific contributions of the muscles necessary to the expression of anger or sadness (e.g. Corrugator). Moreover, it remains unclear if the effect would also apply to the perception of positive emotions.

In the current study, we investigated this issue again, using a unique combination of two different methods.

First, we used a sensitive measure of people’s ability to detect changes in the facial expression exhibited by someone else. Instead of asking people to mentalize or explicitly judge the emotional expression of a static face, or a quickly presented face, we simply asked participants to press a button as soon as they could detect a change in the facial expression exhibited by carefully designed and gradually changing video morphs of an actors’ face continuously and slowly transitioning from a neutral state to an emotional state over 25 seconds. Importantly, in this change-detection task and contrary to previous studies, participants were not asked to explicitly decide about the nature of the change (e.g., recognise happinness), but rather to react with a button-press when they had perceived that the source image had changed. Crucially, it has been reported that detecting a change in the display (instructions focused on the attributes of the image display) involves different neural networks as those required when making an explicit inference about the contents of the change (instructions focused on the nature of the change)^23^. Moreover, the added value of a continuous dynamic change is that it introduces a spatio-temporal continuity not only of the stimulus itself but also of the internal representation of the stimulus^44^. Furthermore, as already demonstrated in our own research dedicated to change blindness^46,48^, participant’s reaction times in this gradual change task offer an implicit, quantitative measure of people’s sensitivity to relevant changes in emotional expression.

Second, to assess how participants responses are affected by the facial musculature, we used BoNT-A so as to directly manipulate people’s ability to mobilize the corrugator supercilli muscle, which is necessary to express anger.

We hypothesised that volunteers treated with BoNT-A would be specifically impaired in the processing of anger. Hence, we predicted that such participants would require more time to detect changes in a slowly and gradually evolving facial expression of anger after treatment (RT post-test > RT pre-test).

## Methods

### Participants

Twelve female volunteers (age range 30–60) were recruited in Brussels and Antwerp from different dermatological clinics. Participants were tested in a quiet room and in dim-light conditions, either at the clinic or at the laboratory. All participants were naïve to any kind of invasive cosmetic procedure, and none were taking medication for the treatment of depression, nor did they present any signs of depression as assessed by the Beck Depression Inventory^49^.

A prior screening was performed to check for a lack of neurological and psychiatric antecedents, as reported verbally by the participants. One participant reported taking medication for high blood pressure (a beta blocker). All subjects presented normal or corrected to normal vision. Participants were not compensated for their taking part in the study, but the cosmetic treatment was performed graciously and all participants were offered a free follow-up consultation. All participants signed an informed consent sheet and were fully informed about the treatment by the dermatologist and by the experimenter, without disclosing the purpose of the study. The study was performed following the guidelines for ethical research and was conducted after obtaining local ethics committee approval from the Faculty of Psychology of the Université Libre de Bruxelles. All subjects were paired in gender, age, and education level to a control group (N = 12), who neither received any treatment nor presented any neurological or psychiatric antecedents.

### Materials

The material consisted of twenty-four stimuli involving gradual facial expression changes created in the laboratory. We asked twelve different actors (4 males and 8 females) to display two different emotions (anger or happiness) and a neutral face. The actors were then photographed with a Canon Ixus 850 IS camera, resulting in a databse of static black and white high resolution pictures (2592 × 1944 pixels). Different morphs that slowly changed from a neutral to full expression of either anger or happiness were then created manually with these pictures. Thus, the scenes containing a facial expression change were based on two different snapshots. To construct the morphs, the expressive face for each actor was first carefully pasted, using Adobe Photoshop CS5 on an exact copy of the neutral expression picture. This yielded three types of pictures, one completely neutral and two other very similar pictures but where the face depicted either a fully expressive face of anger or happiness. Observed pupillary changes have been shown to signal emotional and dispositional changes, and dynamically changing pupils elicit activation of emotional face-related networks (e.g. STS, Amygdala)^50^. Therefore, in order to control for any other social or emotionally relevant changes, the pupilary region of the target image was carefully erased so that only the pupil of the original neutral image was seen throughout the morph. This also ensured that only those pixels related to face morphology and facial expression were changing and thus only information related to the movement of muscle areas could be diagnostic of any change in the display. Subsequently, using morphing software (Morph Man 2000), 625 intermediate frames were assembled in a 25 s sequence in Quick Time format, for a total of 25 images per second with a resolution of 800 × 600 pixels each. From each pair of images (e.g. neutral-full blown anger; neutral-full blown happiness) a movie was then created, one involving a neutral-to-anger change, and the other involving a neutral-to-happiness change.

A total of twenty-four morph videos were then split into two sets of twelve videos each. Each set was then further split into two blocks, each containing 50% of neutral-anger videos, and 50% of neutral-happiness videos. The presentation of the stimuli and blocks was randomised, and each block was repeated only once, for a total of 24 trials per session (12 anger and 12 happiness). Both sets of materials were counterbalanced between sessions (S1 and S2).

Stimulus presentation was programmed in PsyScope and the stimuli were displayed on a 15″ laptop screen at a resolution of 1024 by 768 pixels on a grey background.

### Procedure

Before each testing session (S1 and S2), all participants received a brief ad-hoc checklist created by the experimenter on neurological and psychiatric history, actual medication, alcohol and another stimulant intake, as well as about subjective sleep quality, as it has been shown that sleep disturbances impair correct facial expression processing^51^. None of the participants were excluded following the screening. After a short anamnesis, participants were invited to begin the experiment.

**Figure 1:**
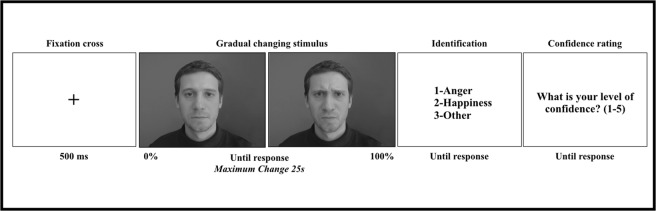
Time course of a single trial. Observers were first exposed to a video clip gradually changing from a neutral expression (0%) to a full blown expression (100%) during 25 s. The stimulus was displayed until observers pressed a button as soon as the change was detected. They were then invited to a forced-choice identification task. Finally, they had to rate the level of confidence of their identification decisions.

After verbal explanation of the instructions, the experimenter left the room, and the participant was left alone. All participants were asked not to cross their legs (e.g this allowed a correct and comfortable sitting posture) and to avoid excessive movements. Participants were instructed that the task consisted of video clips that involved faces that would gradually change over time (Fig. 1). After a fixation cross (500 ms), a video morph was presented. Participants were then instructed to press the button as soon as they could detect a change in the display. Observers were exposed to all changing stimuli until they gave a detection response (button press), after which the video presentation ended. The maximum length of dynamic presentation was 25 s per video, but the presentation continued until the observer responded. This means that it was possible that the observer would “detect” a change only after the video had already reached its full development, in which case the trial was discarded from further analysis.

On each trial, after the detection task, the video presentation terminated and participants were asked to answer two additional questions. First, participants were asked to identify the type of change detected among three possibilities: 1-Anger, 2-Happy, or 3-Other. It was clarified that the option “other” meant something different from either anger or happiness. Finally, participants were asked to rate their confidence in their identification decision on a 5- point Likert scale ranging from 1 (very low confidence) to 5 (very high confidence).

### BoNT-A treatment

The cosmetic treatment took place five to six days after the first computer task (pre-test/S1). The second computer task (post-test/S2) took place on average fifteen days after S1 (BoNT-A: M = 14.8, SD = 1.3; Control: M = 15, SD = 2.3, in days) to ensure maximum toxin relaxation effect in the neuromuscular junction, which usually takes eight to ten days. All participants received a total of 20U of BoNT-A in the frown area, with two to three standard point injections per side (left and right frown area). All volunteers received the same type of toxin. The BoNT-A was diluted with 2.5 ml of physiological serum for every 100 toxin units (Allergan). All participants were injected in the corrugator supercillii muscle, but also in the procerus muscle when necessary (e.g., procerus muscle was too pre-eminent).

At the end of each experimental procedure, change in mood across sessions was assessed by means of the PANAS-now scale^52^ and the State Anxiety questionnaire, STAI-A^53^. All participants were screened for anxiety and depression. Participants in the control group were all tested with a delay between S1 and S2 similar as the delay of the participants in the BoNT-A group to which they were paired.

All statistical analyses were performed on SPSS (IBM SPSS v20.0.0) and JASP (Version 0.8.0) for OS X.

#### Image publication

The authors declare that informed consent has been obtained for permission to publish the images displayed in this manuscript that depicts one of the faces used for stimulus presentation (Fig. 1).

## Results

Unless otherwise specified, all tests were conducted using a two-sided alpha level of 0.05. Trials where a detection response was given beyond the maximum duration of the video morph were discarded. Table 1 shows the percentage of trials discarded per group, session and emotion. *See supplementary information for details*.

**Table 1:** Mean number of error trials discarded from analysis per group, type of change and session based on trials where a detection response time was higher than 25 seconds.

Mean number of Trials discarded for Anger and Happiness (N = 12)				
	S1	S2	SD_S1	SD_S2
BoNT-A-Anger	1	0.17	1.04	0.57
BoNT-A-Happiness	0.25	0.33	0.45	0.49
Control-Anger	1.58	0.42	2.57	0.9
Control-Happiness	0.25	0.08	0.62	0.29

Standard deviations are shown.

### Change detection RT

A three-way 2(Group) × 2(Session) × 2(Emotion) repeated measures mixed ANOVA on log RTs for detection was conducted to understand the effects of frown deafferentation in detection response times of gradual changes of facial expressions. This analysis revealed a significant main effect of Session *F*(1,22) = 6.48, *p* = 0.02, *η2* = *0*.23, but failed to show a significant main effect of Emotion *F*(1,22) = 3,064, *p* = 0.09, *η2* = *0*.12. Moreover, we found no statistically significant three way interaction effect between Group × Session × Emotion, *F*(1,22) = 2,42, *p* = 0.13, *η2* = *0*.10, as well as no significant simple interaction effects between Session × Group, *F*(1,22) = 2.45, *p* = 0.13, *η2* = *0*.10, Emotion × Group, *F*(1,22) = 0.57, *p* = 0.46, *η2* = *0*.03 and Session × Emotion, *F*(1,22) = 0.27, *p* = 0.61, *η2* = *0*.01(see Tables 2 and 3).

**Table 2:** Mean reaction times (*milliseconds*) for the detection of anger at pre-test (S1) and post test (S2).

Mean reaction times for the detection of Anger (ms)			
	Mean	SD	SE
BoNT-A-S1	13010	3287	949
BoNT-A-S2	12982	3328	961
Control-S1	13612	2564	740
Control-S2	10619	3718	1073

Standard deviations and Standard errors are shown.

**Table 3:** Mean reaction times (*milliseconds*) for the detection of happiness at pre-test (S1) and post test (S2).

Mean reaction times for the detection of Happiness (ms)			
	Mean	SD	SE
BoNT-A-S1	12894	2825	816
BoNT-A-S2	11722	3556	1027
Control-S1	11734	2337	674
Control-S2	9780	3712	1071

Standard deviations and Standard errors are shown.

Exploratory direct comparisons were also performed to investigate specific group differences at S2. An independent samples t-test was performed between each group for both emotions at S2. Results failed to show any significant differences between groups, for both, anger, *t*(22) = 1.3, *p* = 0.2, *d* = 0.53, and happiness, *t*(22) = 1.3, *p* = 0.18, *d* = 0.57.

Therefore the full factorial ANOVA was only informative about a main effect of time demonstrating a general decrease in detection RT’s between sessions. However, because we had the a priori hypothesis that deafferentation of the frown area would affect detection times of anger across time in the BoNT-A group, we further tested this prediction by performing Bayesian paired-samples t-tests on Log RT’s of detection for the anger condition between sessions for both groups (*see next section*).

### Bayesian analysis on detection RT for the BoNT-A group

Frequentist statistics can only tell us that an alternative hypothesis was not supported while being mute on whether the data truly support the null (i.e. the compared means are truly not different) or whether the design was insensitive^54,55^. For instance, a non significant p-value cannot be interpreted as the absence of evidence for a difference between measures, nor does it mean that we have evidence of an absence of difference^56^. The Bayes Factor (B) is a measure of how well our data can be predicted by the alternative hypothesis (H1) or how well it is best explained by the null (H0). A calculated B-value of 3 or more indicates evidence in favour of H1, a B-value of 1/3 or below indicates evidence for the null and a value between 1/3 and 3 is indicative of insensitivity, meaning that one cannot favour either hypothesis, or one has “no evidence to speak of”^57^.

Our alternative hypothesis (H1) stated that reaction times at S2 for anger are higher than S1 because we predicted that the BoNT-A treatment would affect reaction times only for the detection of anger. This a priori prediction, however, could not be supported by our analyses. We can therefore ask if our data was not sensitive or wether there truly was no difference between S1 and S2 for anger in the BoNT-A group. To find out, we performed a Bayesian paired samples t-test on RT’s for the detection of anger for the BoNT-A group. As our B is close to 0 (BF10 = 0.25), we express the results as a function of the Null (BF01). The Bayes factor (BF01) was B = 4.05, which means that our data are 4 times more likely under the null than under the alternative. Hence, we can conclude that we have moderate evidence in favour of the null. This suggest that our data does not support the theory that reaction times at S2 are higher than reaction times at S1 in the BoNT-A group, and our results are hence best explained by an absence of difference between S1 and S2 for that condition.

**Figure 2:**
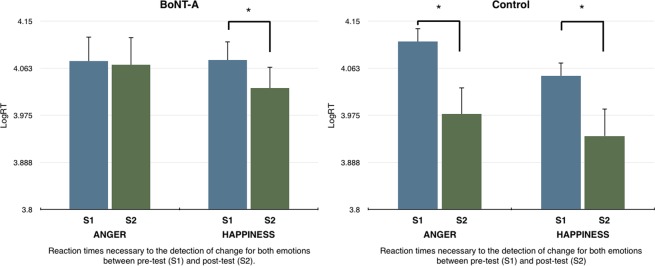
Reaction times for detection of both emotions at pre-test and post-test. *Error bars represent standard error (N* = *12 for each group)*.

Because the full factorial ANOVA only informed about a general decrease in reaction times between sessions, we performed a similar Bayes Factor analysis to assess the amount of evidence supporting an improvement for the control group in the detection of Anger. Considering that this alternative hypothesis (H1) is that there is a difference (RT at S1 > RT S2), the Bayes Factor for reaction times of detection of Anger is BF10 = 4.55. We can, therefore, conclude that we have moderate evidence in favour of the hypothesis that the control group did improve from S1 to S2 in the detection of Anger. *A frequentist statistics equivalent of the analyses were performed* (Fig. 2); *details are shown in the supplementary information section*.

### Identification and confidence RT

To analyse reaction times in the identification task and in the confidence rating task, we performed a Group (2) × Session (2) × Emotion (2) repeated measures mixed ANOVA on log RT’s of identification responses and of confidence rating responses. We analysed data from the trials that were included in the analyses following the same time constraint as for detection, that is, trials for which a response had been provided before the video had lapsed (i.e., after 25 seconds). Results of both analyses revealed a significant main effect of session (all *p*’s < 0.005). Furthermore, the analyses for the RT’s of confidence ratings also revealed a main effect of Emotion (*p* = 0.018) showing that overall RT’s of confidence for identification responses of changes of happiness were smaller than for anger. This suggests a general decrease in identification as well as confidence judgment RT’s between sessions, and that confidence judgments for identifications of anger take longer times than for happiness. *For details see supplementary information*.

### Identification accuracy

In order to analyse accuracy, A full factorial 2(Group) × 2(Session) × 2(Emotion) repeated measures mixed ANOVA on percentage of correct responses was conducted revealing a significant main effect of Session *F*(1,22) = 4,72, *p* = 0.04, *η2* = 0.18 and a significant main effect of Emotion *F*(1,22) = 16.78, *p < *0.001, *η2* = 0.43. The three-way interaction effect, Group × Session × Emotion was not significant *F*(1,22) = 0.06, *p* = 0.80, *η2* = 0.003, and none of the simple interaction effects reached significance either, Session × Group, *F*(1,22) = 0.04, *p* = 0.83, *η2* = 0.002, Emotion × Group, *F*(1,22) = 0.09, *p* = 0.77, *η2* = *0*.004 and Session × Emotion, *F*(1,22) = 1.97, *p* = 0.18, *η2* = 0.08. This suggests a general improvement in identification accuracy from S1 to S2. Moreover, all participants were more accurate when identifying changes of happines at both sessions (see Table 4).

**Table 4:** Mean percent accuracy for the identification of both emotions by group at pre-test(S1) and post-test (S2).

Mean Accuracy (%) for Anger and Happiness				
	S1	S2	SD_S1	SD_S2
BoNT-A-Anger	0.69	0.78	0.22	0.20
BoNT-A-Happiness	0.89	0.93	0.23	0.12
Control-Anger	0.68	0.76	0.22	0.18
Control-Happiness	0.86	0.88	0.17	0.09

Standard deviations for both sessions are shown.

### Confidence ratings

To assess confidence ratings on a trial-by-trial basis, participants were asked, after identification response, to express how confident they were about their decisions (on a Likert scale ranging from 1 to 5). Our aim was to gauge the extent to which participants were sensitive to their own correct or incorrect decisions (metacognitive accuracy, see ref.^58^). Because these results did not yield interpretable effects, they are only reported in the supplementary information section.

### Questionnaires and mood change congruency

In order to gauge any change in mood between sessions, as well as to assess any linear relationship between mood change and its influence on detection RTs, participants were administered the Positive and Negative Affect Schedule PANAS^52^ scale and the Spielberger State Anxiety questionnaire, STAI-A^53^ after the experiment had been completed, once before and once after treatment.

For the BoNT-A group, a paired-samples t-test on the PANAS Negative scores observed before and after treatment was not significant across sessions, *t*(11) = 1.925, *p* = 0.08, *d* = 0.56, (Before: M = 19, SD = 7; After: M = 17, SD = 6). Results for STAI-A scores similarly failed to reveal a difference before and after treatment, *t*(11) = 2.104, *p* = 0.059, *d* = 0.6 (Before: M = 40, SD = 10; After: M = 35, SD = 10). No difference was found either for the PANAS Positive scores between sessions, *t*(11) = −0.67, *p* = 0.514 *d* = 0.19, (Before: M = 33, SD = 9; After: M = 34, SD = 7). This same analysis performed for the Control group likewise showed no difference before and after treatment, neither for Positive *t*(11) = −0.25, *p* = 0.80, *d* = 0.07, (Before: M = 34, SD = 7; After: M = 34, SD = 4) or Negative *t*(11) = 1.54, *p* = 0.15, *d* = 0.44, (Before: M = 18, SD = 5; After: M = 16, SD = 6) PANAS scores, nor for STAI_A scores *t*(11) = −0.24, *p* = 0.81, *d* = 0.07, (Before: M = 34, SD = 7; After: M = 35, SD = 10).

We then performed a correlational analysis on the change in RT before treatment versus after treatment, and the change in PANAS scores for each subscale (Session 2 minus Session 1). Analysis on the BoNT-A group failed to show any significant relationship between change variables. However, results on the Control group showed a significant moderate negative correlation *r* = −0.579, *p* = 0.048 between change in positive affect and RT change for the detection of anger, and a marginally significant negative correlation (moderate), *r* = −0.570, *p* = 0.053 between change in positive affect and RT change for the detection of happiness. Essentially, these results highlight a relationship between an increase in positive affect at Session 2 (S1:33, SD = 9; S2:M = 34, SD = 7) that is significantly correlated to a decrease in RT in Session 2, compared to Session 1, as participants become faster overall (*see above*).

## Discussion

The aim of the present study was to test how paralysis of the frown area of the face affects the processing of anger. We predicted that frown paralysis would result in higher reaction times and decreased accuracy to detect gradual changes involving anger.

In line with our hypothesis, we found that only the BoNT-A group showed a differential pattern of responses. All participants showed shorter response times in all three tasks in the post-test compared with the pre-test. However, further Bayesian analysis demonstrates that this improvement was absent in the BoNT-A group when detecting changes of anger. We interpret the lack of improvement specific to the BoNT-A group in S2 as evidence of a disadvantage caused by deafferentation, and further suggest that our RT-based measure of gradual change detection was more sensitive than traditional accuracy-based measures.

### Facial feedback is muscle specific and differentially contributes to face processing depending on task demands

Our findings support the idea that specific facial actions influence the perception of those same facial changes. This clarifies former research showing heterogenous results, most likely attributable to the fact that several muscles had been compromised in these studies (i.e., BoNT-A was applied in several areas). To our knowledge, the only study assessing face processing and specifically targetting the frown area^43^ failed to show any behavioural effects of deafferentation. With our paradigm, we were able to demonstrate a specific effect in regards to anger processing.

Furthermore, during detection, a different mechanism was at play. We reason that it is more likely that perceptual processes that were recruited during the detection task were the ones that were primarily modulated by deafferentation but not those that are related to more elaborate (e.g. semantically loaded) processing. This interpretation is in accordance with accounts that posit two separate routes of face processing, one dedicated to semantically loaded face information (e.g. recognition system/identification system) and a parallel and independent one, not experience-dependent, relative to detection of face stimuli, which relies primarily on exogenous attention (i.e. bottom-up driven processing)^59^. It could be argued that evaluative (e.g. conceptual) processes were unavoidably affected, for instance, because participants might have been trying to constantly guess the nature of the upcoming change. However, even if participants could guess the nature of the change they had to detect, changes were contingent on stimulus characteristics, which were seemingly not perceived faster in the case of anger. As such, if participants would have acted solely based on knowledge of the valence of the stimuli, they could have performed the detection task based on mere discrimination of the items, particularly because happiness was always easier to detect (e.g. “since it is not happiness it must be anger”). If this was the case, the BoNT-A group would have presented a similar pattern of detection responses for anger at session 2. However, our results did not confirm this.

#### Anger vs. Happiness

With respect to the distinction between face conditions, we believe that a simulation^18^ process based on “motor-matching” rather than on “affective-matching”, such as the mirror neuron system, can account for the difference between anger and happiness. Both facial expressions are not only perceptually different, but their associated motor plans and production depend on distinct dedicated neuroanatomical structures^60^. Further support comes from the study by Tamietto *et al*.^17^. In their study, they showed that a cortically blind patient presents spontaneous and congruent facial reactions to emotional faces and bodies as assessed with EMG. The fact that these effects were observed irrespective of the type of stimuli was interpreted as supporting the hypothesis that the patient resonated with the affective meaning (i.e.concepts) rather than with motor-matching. This was suggested to take place via a route that bypasses the visual cortex through the superior colliculus and pulvinar, and that enables the affective evaluation of the stimuli. Ultimately, this nonconscious path helps the cortically blind patient to perform at above chance-level in an emotion recognition task. However, these congruent EMG responses were slower, yet stronger for faces than for bodies when the stimuli were consciously perceived, suggesting that a motor resonance mechanism was at play and that it depends on perceptual awareness and on cortical vision. As such, while it cannot be ruled out that evaluative affective processes must have been triggered during exposure to facial changes in our present study (and most likely this was the case), what we observe is that participants could not perceive a specific change faster. Therefore, because participants were fully aware, and because changes were highly visible, it seems more likely that it was a perceptual decision that was modulated and not a decision based on affective evaluations per se, at least not to the extent that perceptual processes were also modulated, in which case we should have observed that the identification task or confidence level ratings were also modulated. Crucially, correlational analyses for each group and each emotion between reaction times for detection and reaction times for identification and confidence rating respectively, failed to show any linear associations at both sessions (all *p*’s > 0.1, see Tables S7 and S8 supp. Info.).

Note, however, that across sessions, a significant correlation was observed in the control group only (see Tables S7 and S8 supp. Info.), showing that identification times of either emotion at S2 were linearly related to previous detection performance of happiness changes at S1. It is, therefore possible that participants’ response times during identification and confidence ratings at S2 relied on their previous experience with the material for which they had faster and better responses at S1. This suggests that BoNT-A may have further modulated the involvement of memory processes and experience-dependent mechanisms at S2 during identification and confidence ratings as well.

### Face paralysis affects detection improvement

Our results concerning the lack of improvement for the BoNT-A group is suggestive of an implication of facial feedback in learning. As in previous studies using BoNT-A (e.g.^32–35^), we expected that the time for change detection of anger would increase. However, even though participants were exposed to a completely different material at post-test, they exhibited an advantage, but this advantage did not favour the BoNT-A group in the detection of anger. This unexpected improvement at S2 is in line with research on memory dynamics using gradually morphed faces evolving from a familiar face (source) towards an unfamiliar face (target), showing that after repeated exposure of the morph, an increasing fraction of the morph is identified as the source face (familiar) across time. However, this happens only if changes within the morph occur in a gradual increasing manner (vs. mixed order)^61^. In other words, strong attractor representations can result from repeated exposure to gradual increasing changes and in such situations, the representations of the morph show strong convergence between source and target. This predicts a decrease in discriminability between source and target^62^. It is thus possible that in our paradigm, the nature of the dynamic stimuli promoted a history-dependent effect, where, first, re-exposure of a similar pattern of changes resulted in a unified memory representation of visual features changes, and second, because of this the introduction of a completely different set of faces at S2 made it possible to extract distinctive facial features more accurately. Crucially, it has been shown that detecting changes in such paradigms depends more on short-term memory representations than on long-term semantic knowledge^44–46^. The only study using BoNT-A that specifically targeted the corrugator muscle and gauged paralysis effects on perceptual decisions^43^ did not find any behavioral results, most likely because, after each target, a 250 ms mask appeared in order to create “retinal wipe” and target presentation times were too short (17 ms, 50 ms, and 1000 ms). In our paradigm, visual changes not only lasted several seconds, but were continuous, dynamic, and increased over time. Furthermore, dynamic facial changes compared to static faces are known to promote processing and representation enhancement, at the behavioral and neural level^63,64^. Interestingly, familiarity after -non-reinforced- repeated exposure to faces, has been shown to increase zygomaticus (but not corrugator) muscular activity at re-exposure^65^. Even though our results concern the corrugator muscle, an affective-perceptual fluency account could further explain these results^66^. As the stimuli became more familiar, both types of stimuli result perceptually more fluent, producing overall faster responses at S2.

As such, our findings are suggestive of disrupted associations between perceptual cues and somatosensory information. In keeping with this interpretation, encoding and retrieval of emotional words has been shown to benefit from motor mimicry^67^. In a similar manner, it is possible that subjects in our study failed to make use of such associations which resulted in a clear lack of improvement in the threshold to detect anger.

### Face paralysis and information processing: Is it a question of time or a question of the amount of information?

The fact the BoNT-A group did not become faster when processing anger changes implies they might have needed more information (percentage of face change of the stimulus) before detection, contrary to what was observed when they detected changes of happiness, or in the control group. If the stimulus would have been static, we could argue that the processing of the emotional stimulus simply needed more time. However, since our stimuli were dynamic and continuously changing, the amount of information increased accordingly, and so did the visual representation of the stimulus^44^. It is thus difficult to disentangle a slow-down in the processing from the need of more salient information. Importantly, reporting what the change is about, where or when the change happens or if a change happens at all, all constitute different aspects of change perception and are constrained by different attentional and memory capacities^68^. Research that used flicker paradigms, however, has shown that independently of task complexity, iconic memory traces act as surrogates, just as if a previously presented stimulus was still visible^69^. Interestingly, the use of continuous flash suppression (C-FS), demonstrates that presentation of congruent proprioceptive information can modulate awareness of the suppressed image. However, this has not been observed when stimuli are emotional faces and mimicry has been probed (eg. biting a pen)^70^. The study by Korb et al. did not find evidence of spontaneous facial mimicry on all trials as assessed by EMG, perhaps due to visual memory constraints created by the rapidly suppressing stimulus. This is a limitation that a constantly changing stimulus might overcome. Hence, detection of change is contingent on the comparison of a contiguous structure stored in memory. It has been established that facial information can quickly bypass the visual cortex^71^, reach limbic structures and elicit mimicry^72^. Interestingly, while processing faces, the visual cortex receives input as top-down modulations from these same regions (e.g., amygdala)^73^ and deafferentation with BoNT-A seem to selectively attenuate their activity^37,43^. Therefore, a testable hypothesis is that if face deafferentation affects the sensitivity of these emotional centers to faces, input from them to the visual cortex should be affected as well. A facial feedback response, in this case, could act as an error-correction process^74^, and the discrepancy between both percepts might not be resolved until a salient change is perceived. Therefore the comparison of a representation in memory against the representation of a currently visible one might be difficult, resulting in a phenomenon similar to change blindness^75^. Literature shows how updates of visual memory in change detection tasks do not necessarily require explicit detection of the change and that updates of visual memory happen in an incremental fashion as well^45^.

### Short-term and long term effects of deafferentation

A difficult issue in the study of deafferentation effects, is to know if the results are due to the general effect of deafferentation, that is, the fact that participants could not frown at all, or to the impossibility for them to engage in mimicry during the task. It is important to note that the specific action of BoNT-A is to impede the release of Acetylcholine (AcH) into the neuromuscular junction^28^, thus preventing the contraction itself. This blockage dampens the afferent signal to the CNS^76^. Thus, the missing signal is the afferent feedback signal, not the efferent signal that commands mimicry per se, which remains unaltered after BoNT-A injections^29^. In this respect, it remains difficult to assess if it was feedback mediated through mimicry that was the specific cause of these effects. A simulationist account^18^ would imply that this was indeed the case, most probably through proprioception. Importantly, while the traditional view is that a dedicated muscular proprioceptive mechanism of the face does not exist^60,77^,recent findings demonstrate that structures similar to the Ruffini corpuscles, one of the two major mechanoreceptors, can be found in deep layers of the facial musculature^78^.

On the other hand, deafferentation of frown muscles promotes a functional reorganisation of key emotional centres^37,43^. These findings are in line with observations in patients presenting muscular dystonias^29^, for which the use of BoNT-A to alleviate their symptoms produces a reorganisation of abnormal cortical maps as well as a change in cortical plasticity^79^. Such changes occur in a short period of time of only 7 days after deafferentation^80^. In our study, the post-test took place between 8 and 10 days after paralysis, which allows for sufficient time to enable a functional reorganisation of somatosensory cortical centres. This in line with the observation that virtual lesions with TMS to the somatosensory face region is sufficient to affect the perception of emotional changes from anger to happiness^22^ and judgements of amusement from faces^23^. However, transient neural noise cannot be observed as functional reorganisation and different mechanisms are at play. Further understanding of these cortical dynamic changes can help clarify why the participants in our study could not improve at S2.

### Social aspects of facial paralysis and mood congruency

It is important to note that an alternative explanation to our results might be related to a loss of sensitivity either through a habituation effect or a coping strategy due to the changes of face schema^81^. It has recently been shown that while facial disfigurement usually elicits disgust in the observer, patients are insensitive to it either because of habituation to the emotion elicited in others or as a protective mechanism from being rejected by peers. In a similar way, it is possible that BoNT-A participants were sensitive to any change expressed by their entourage after the cosmetic treatment, for instance, that their peers might have found the aesthetic change unfamiliar and therefore disfluent/odd. Moreover, participants in our study could as well have engaged in a coping mechanism, therefore devoting less attention to negative stimuli as a protection against the prospect of social rejection. Facial expressions are key features of human communication, and as such the social aspects (e.g. expectations) of aesthetic change cannot be disregarded.

In keeping with these considerations, note that only the BoNT-A group showed a decrease in both negative affect and in state anxiety scores, although only at a trend level (*p* = 0.08 and *p* = 0.059 respectively). This stands in contrast to what was reported by Havas *et al*.^32^, the only study specifically targeting the frown area, and which demonstrated an effect on negative emotional processing. In their study, BoNT-A participants showed a significant decrease in positive affect (*p* = 0.01), but no such effect relative to negative affect scores (p = 0.625). The authors interpreted this as sufficient evidence to rule out mood congruency, with no explanation of the significant decrease in positive affect scores. Similarly, Davis *et al*.^35^ reported a decrease in positive affect and an increase in negative affect. We believe these results must, however, be interpreted with caution, because either BoNT-A was injected in several regions at the same time^35^ or toxin units (TU) were not the same for all participants^32^, as was the case in our study. Our results suggest the possibility of an overall mood-congruent effect that is consistent with a protective account (e.g. less attention to negative emotions) and further supports findings related to the remission from mood disorders^39,40^ after BoNT-A injections in the glabella. The fact that the decrease in negative affect was only found at a trend level warrants for caution and suggests that further research is needed to clarify these results.

Finally, an interesting finding was the significant negative correlation between change in positive affect and reaction time. However, this was observed only in the control group for whom the higher the score in positive affect change, the shorter the reaction time for the detection of either emotion, although only at a marginal level for anger (*p* = 0.053). This suggests that a reward-based mechanism might have been at play during detection response, a hypothesis compatible with the notion that an optimal action-selection can be predicted if the reward feedback related to it can be properly simulated^32,82^. This view also in line with our previous interpretation of an affective-perceptual fluency account^66^. Therefore, it is possible that because of the BoNT-A action on CNS^37,43^, optimal action selection did not properly succeed given the drop in action-value functions^82^. Crucially these results suggest that although the results for happiness detection seemed immune to frown deafferentation, the relationship between subjective experience of positive affect and detection performance might have also been modulated. This further supports the view that deafferentation did modulate the BoNT-A group’s performance and highlights the fact that positive and negative emotional processing are interdependent processes. Future work using functional imaging and connectivity analyses could help clarify the involvement and relationship between value centres (e.g. Amygdala) and reward systems (e.g. striatum) in situations of frown deafferentation and emotional processing.

### Limitations

A limitation of this study is the fact that our control group did not receive any treatment. While a placebo control (e.g., a saline solution) would indeed have been more specific, the use of an A-B design is such that each group acts as its own control. It is worth noting that the literature contains both instances of studies that have appealed to alternative treatments (e.g., fillers, see refs^34,35^) and instances of studies that do not (see refs^32,43^). With our design, we not only have information about intra-group differences but also about inter-group differences. One may still argue that we cannot prove that the effects we observe specifically depend on the administration of BoNT-A versus a placebo. Thus, it is not impossible that we would have observed the same pattern of results in a group of participants administered a saline solution in the same area of the face. While this would be interesting in and of itself as a study on placebo-induced beliefs, we very much doubt that we would have observed the specific disadvantage in detecting anger, particularly given that the effects we observed were measured indirectly and implicitly. Another limitation is the fact that participants consistently responded with the same hand and the same order of buttons, which might partly explain why they became quicker overall at S2. However, the observation that both groups became significantly more accurate and faster at S2 shows that their performance is congruent with effective processing and not a time-accuracy trade-off. Thus, the effects we observed seem to be specific to the change detection task. With respect to our methods, an important limitation was that despite the fact that special care was taken to create the video-morphs as accurately as possible (e.g., high amount of corresponding transition points, ad hoc face delineation for each model), some of the stimuli nevertheless exhibited some image imperfections (e.g., “ghosts”). We do not believe, however, that this limitation is a major issue because we successfully used the same algorithm and software to create complex material for a similar gradual changes paradigm in which we observed robust change blindness effects^46^.

Finally, even though we did not have an explicit hypothesis concerning confidence ratings, it could have been of interest to assess the effects of deafferentation on metacognition, which is an indirect measure of subjective experience. However, despite the fact that confidence ratings are the most exhaustive measures of subjective experience of emotional stimuli^83^, we used a 5-point scale, which made it difficult to calibrate in either a dichotomous (e.g. guess vs. know) or a continuous scale (e.g. high/low on a 4-point scale**)**^84^. Future research should help clarify this issue with the use of better tools to assess subjective reports.

## Conclusions

Understanding biosocial events rely on an accumulation of perceptual information and involve interactions between neural systems for action and perception^85^. With our paradigm, we contribute to elucidate how Facial Feedback is causally effective at processing gradual changes of emotional facial displays. Such effects may be well supported by a system that has learned to anticipate future occurrences of a given representation^86^ on the basis of perceptual and somatosensory associations. The contributions of face proprioception to consciousness have been challenged^70^, therefore a critical step forward would be to clarify the role of facial feedback over different time scales and how facial sensorimotor associations develop in concert with the putative cortical and subcortical systems that support them. Future research in this direction might help better understand the scope of clinical uses of facial deafferentation.

## Supplementary information

Supplementary Information

## Data Availability

Example of stimuli materials and data supporting the findings of the current study can be found here: https://osf.io/q7dtp/?view_only=c7331f980bcd4c1abe370ae7e6fcd82d.
