# Supplementary material for: The effects of Botulinum toxin on the detection of gradual changes in facial emotion
Source: Sci Rep. 2019 Aug 13;9:11734. doi: 10.1038/s41598-019-48275-1 (PMC6692314; doi:10.1038/s41598-019-48275-1)
Supplement: Supplementary file 1 — Supplementary Information [file 41598_2019_48275_MOESM1_ESM.pdf]

Nature Scientific Reports,

Supplementary information
"The effects of Botulinum toxin on the detection of gradual changes in facial emotion"

Luis C. Bulnes, Peter Mariën, Marie Vandekerckhove, Axel Cleeremans.

Supplementary information

Mean Time Accuracy (%) for **Anger and Happiness**				
	**S1**	**S2**	**SD_S1**	**SD_S2**
BoNT-A-Anger	0.92	0.98	0.09	0.05
BoNT-A-Happiness	0.98	0.97	0.04	0.04
Control-Anger	0.87	0.96	0.21	0.07
Control-Happiness	0.98	0.99	0.05	0.02

Table S1_related to Table 1. Mean percentage of trials used for analysis per group, type of change and session based on trials with a detection response time not higher than 25 seconds. Standard deviations are shown. When a detection response exceeded 25s of reaction time, the trial was discarded for analyses. Only responses given based on time accuracy were retained.

Total number of trials discarded for **Anger and Happiness (Nt=144 per cell)**				
	**Anger S1**	**Happiness S1**	**Anger S2**	**Happiness S2**
BoNT-A	12	3	2	4
Control	19	3	5	1

Table S2_related to Table 1. Total number of trials discarded per group, type of change and session.

Frequentist equivalent of a priori hypothesis testing for detection RT

Note that the frequentist equivalent yield similar results as the Bayesian analysis. In order to accurately calculate this we adjusted the error term as estimated from the error term (MS-error) of the factor Session from the overall ANOVA. Contrary to what we expected, this analysis failed to show any difference between sessions for the BoNT-A group, $t(11) = 0.29$, $p = 0.78$, $d = 0.063$. The same analysis, however, revealed a significant difference between sessions in RTs for the detection of anger in the control group, $t(11) = 6.136$, $p < 0.0001$, $d = 0.71$, (Fig. 2) . The Control group showed shorter reaction times in detecting an emotional change of anger at S2 compared to S1.

Identification and confidence RT

A full factorial 2(Group) x 2(Session) x 2(Emotion) repeated measures ANOVA on log RTs for identification was conducted revealing a significant main effect of Session $F(1,22) = 14.85$, $p = 0.001$, $\eta 2 = 0.403$ but failed to show a significant main effect of Emotion $F(1,22) = 0.001$, $p = 0.97$, $\eta 2 = 0.00$, as well as a tripple interaction effect, Group x Session x Emotion, $F(1,22) = 0.06$, $p = 0.80$, $\eta 2 = 0.003$. None of the simple interaction effects reached significance either, Session x Group, $F(1,22) = 1.43$, $p = 0.24$, $\eta 2 = 0.06$, Emotion x Group, $F(1,22) = 0.83$, $p = 0.37$, $\eta 2 = 0.04$ and Session x Emotion, $F(1,22) = 0.31$, $p = 0.57$, $\eta 2 = 0.01$.

A series of similar analyses were performed to analyse reaction times of the confidence ratings task.

A full factorial 2(Group) x 2(Session) x 2(Emotion) repeated measures ANOVA on log RTs for confidence ratings was conducted revealing a significant main effect of Session $F(1,22) = 17.02$, $p < 0.001$, $\eta 2 = 0.43$ and a

significant main effect of Emotion, $F(1,22) = 6.53$, $p = 0.018$, $\eta 2 = 0.23$. The tripple interaction effect, Group x Session x Emotion was not significant $F(1,22) = 0.35$, $p = 0.56$, $\eta 2 = 0.02$. None of the simple interaction effects reached significance either, Session x Group, $F(1,22) = 0.63$, $p = 0.43$, $\eta 2 = 0.03$, Emotion x Group, $F(1,22) = 3.64$, $p = 0.069$, $\eta 2 = 0.14$ and Session x Emotion, $F(1,22) = 0.65$, $p = 0.80$, $\eta 2 = 0.03$.

. See tables S3-S6.

Mean Log RTs for the identification of **Anger**		
	**Mean**	**SD**
BoNT-A-S1	3.40	0.21
BoNT-A-S2	3.31	0.25
Control-S1	3.30	0.18
Control-S2	3.13	0.32

Table S3. Mean reaction times (Log RT) and standard deviations for the identification responses of anger.

Mean Log RTs for the identification of **Happiness**		
	**Mean**	**SD**
BoNT-A-S1	3.38	0.24
BoNT-A-S2	3.30	0.23
Control-S1	3.31	0.21
Control-S2	3.23	0.24

Table S4. Mean reaction times (Log RT) and standard deviations for the identification of happiness.

Mean Log RTs for confidence ratings of **Anger**		
	**Mean**	**SD**
BoNT-A-S1	3.28	0.15
BoNT-A-S2	3.19	0.24
Control-S1	3.20	0.25
Control-S2	3.05	0.28

Table S5. Mean reaction times (Log RT) and standard deviations for the confidence ratings over identification of anger.

Mean Log RTs for confidence ratings of **Happiness**		
	**Mean**	**SD**
BoNT-A-S1	3.20	0.26
BoNT-A-S2	3.09	0.30
Control-S1	3.18	0.25
Control-S2	3.04	0.30

Table S6. Mean reaction times (Log RT) and standard deviations for the confidence ratings over identification of happiness.

Correlational Analyses between reaction times (RT's) for Detection, Identification and Confidence rating.

Table S7. Correlations between reaction times (Log RT's) for all three tasks for the **BoNT-A group**. The grayed area shows the critical correlations between detection RT's and RT's for both subsequent tasks (identification and confidence rating).

Correlations between reaction times for detection and subsequent identification and confidence rating reaction times for the ***BoNT-A group*** *(N=12).*

Variables	1	2	3	4	5	6	7	8	9	10	11
1. RT detection Anger S1	-										
2. RT detection Happy S1	0.351	-									
3. RT detection Anger S2	.826**	0.324	-								
4. RT detection Happy S2	0.369	0.492	.659*	-							
5. RT Identification Anger S1	0.024	0.063	0.316	0.229	-						
6. RT Identification Happy S1	-0.032	0.025	0.143	0.129	.759**	-					
7. RT Identification Anger S2	-0.329	-0.135	-0.046	-0.004	.752**	.755**	-				
8. RT Identification Happy S2	-0.302	-0.154	-0.102	-0.031	.701*	.870**	.900**	-			
9. RT Confidence Anger S1	-0.024	-0.213	0.041	-0.106	.587*	.751**	.587*	.741**	-		
10. RT Confidence Happy S1	0.023	-0.127	0.078	-0.011	0.512	.802**	.749**	.829**	.854**	-	
11. RT Confidence Anger S2	-0.231	-0.421	-0.104	-0.14	0.421	.616*	.675*	.827**	.799**	.815**	-
12. RT Confidence Happy S2	0.024	-0.274	0.011	-0.033	0.146	0.524	0.401	.658*	.674*	.761**	.894**

**p*< .05. ***p*< .01. ****p*<0.001.

Table S8. Correlations between reaction times (Log RT's) for all three tasks for the **Control group**. The grayed area shows the critical correlations between detection RT's and RT's for both subsequent tasks (identification and confidence rating).

Correlations between reaction times for detection and subsequent identification and confidence rating reaction times for the ***Control group*** *(N=12).*

Variables	1	2	3	4	5	6	7	8	9	10	11
1. RT detection Anger S1	-										
2. RT detection Happy S1	0.479	-									
3. RT detection Anger S2	-0.015	0.084	-								
4. RT detection Happy S2	0.077	0.092	.956**	-							
5. RT Identification Anger S1	-0.06	0.376	0.464	0.333	-						
6. RT Identification Happy S1	-0.276	0.305	0.431	0.343	.851**	-					
7. RT Identification Anger S2	-0.127	**.666***	0.144	0.049	.756**	.794**	-				
8. RT Identification Happy S2	-0.077	**.628***	0.114	-0.013	.787**	.805**	.930**	-			
9. RT Confidence Anger S1	0.165	0.393	0.298	0.216	0.476	.702*	0.52	.596*	-		
10. RT Confidence Happy S1	-0.016	0.486	0.567	0.481	0.504	.685*	0.565	0.538	.825**	-	
11. RT Confidence Anger S2	0.074	0.536	0.041	-0.049	0.345	0.568	.590*	.614*	.856**	.803**	-
12. RT Confidence Happy S2	0.098	0.468	0.052	-0.037	0.266	0.515	0.49	0.533	.901**	.786**	.951**

**p*< .05. ***p*< .01. ****p*<0.001.

Identification Accuracy

Mean Identification Accuracy (%) for **Anger and Happiness**				
	**S1**	**S2**	**SD_S1**	**SD_S2**
BoNT-A-Anger	0.69	0.78	0.21	0.24
BoNT-A-Happiness	0.89	0.93	0.22	0.12
Control-Anger	0.68	0.76	0.22	0.18
Control-Happiness	0.87	0.88	0.17	0.09

Table S9. Mean percentage of trials where a correct identification was given, per group, type of change and session. Standard deviations are shown.

Confidence ratings

Gamma correlations were computed to gauge "relative meta-accuracy" that is, the extent to which participants are able to discriminate between their own correct and incorrect decisions. Gamma correlations are non-parametric correlation coefficients that relate individual trial-by-trial confidence ratings and correct versus incorrect responses (1 or 0) in the identification task.

To determine if confidence level differed overall, we performed paired samples t-tests for each group between each session for both types of changes. All confidence ratings were transformed into proportions. This analysis failed to show any significant difference between S1 and S2 for both types of changes (all p-values > 0.3). We then proceeded to the assessment of relative meta-accuracy. One constraint of the analysis is that participants need to show variability in their first-order accuracy and in their second-order confidence judgments. In this respect, given that participants where highly accurate identifying changes of happiness, and that most participants reported being highly confident in these discriminations, no gamma correlations are reported for happiness.

Thus, we report gamma correlations for the detection of anger only for those participants for whom it was possible to compute the index. Unfortunately, for the BoNT-A group, gamma correlations could only be computed for 8 participants, whereas for the control group, it was possible only for 9. These gamma correlations (G) where first compared to zero, so as to first assess if participants were better than chance at discriminating correct vs. incorrect decisions. Interestingly, while at S1, for the BoNT-A group, G is not significantly greater than zero, $t(7) = 1.55$, $p = 0.165$, at S2, results show a significant difference compared to the null, $t(7) = 2.93$, $p = 0.022$. By contrast, for the control group, we find a difference between G and zero at S1, $t(8) = 5.06$, $p = 0.001$; however this difference is no longer significant at S2, $t(8) = 0.862$, $p = 0.414$. Further analysis focused on differences in G between sessions for each group. This yielded no significant difference for the BoNT-A group,

$t(8) = -1.29$, $p = 0.24$, $d = 0.45$, and revealed only a trend for the control group, $t(9) = 1.89$, $p = 0.095$, $d = 0.63$, where gamma correlations are smaller at S2 (M=0.22,SD=0.75) compared to S1(M=0.59, SD=0.35). These results suggest that not only did both groups of participants remain equally confident of their identification decisions across time, but also that the BoNT-A treatment had no effects on how sensitive participants were to their accuracy on a trial-by trial basis at S2 compared to S1.
